# Mobile Phone Use in Psychiatry Residents in the United States: Multisite Cross-Sectional Survey Study

**DOI:** 10.2196/mhealth.7146

**Published:** 2017-11-01

**Authors:** Shih Gipson, John Torous, Robert Boland, Erich Conrad

**Affiliations:** ^1^ Department of Psychiatry Boston Children's Hospital Boston, MA United States; ^2^ Department of Psychiatry Harvard Medical School Boston, MA United States; ^3^ Department of Psychiatry Beth Israel Deaconess Medical Center Boston, MA United States; ^4^ Department of Psychiatry Brigham and Women's Hospital Boston, MA United States; ^5^ Department of Psychiatry Louisiana State University Health Sciences Center New Orleans, LA United States

**Keywords:** technology, graduate medical education, mobile phone, psychiatry

## Abstract

**Background:**

Mobile technology ownership in the general US population and medical professionals is increasing, leading to increased use in clinical settings. However, data on use of mobile technology by psychiatry residents remain unclear.

**Objective:**

In this study, our aim was to provide data on how psychiatric residents use mobile phones in their clinical education as well as barriers relating to technology use.

**Methods:**

An anonymous, multisite survey was given to psychiatry residents in 2 regions in the United States, including New Orleans and Boston, to understand their technology use.

**Results:**

All participants owned mobile phones, and 79% (54/68) used them to access patient information. The majority do not use mobile phones to implement pharmacotherapy (62%, 42/68) or psychotherapy plans (90%, 61/68). The top 3 barriers to using mobile technology in clinical care were privacy concerns (56%, 38/68), lack of clinical guidance (40%, 27/68), and lack of evidence (29%, 20/68).

**Conclusions:**

We conclude that developing a technology curriculum and engaging in research could address these barriers to using mobile phones in clinical practice.

## Introduction

Mobile technology ownership is common in the United States with 77% of the population owning a mobile phone [1]. In learning and in practice, medical students and trainees also have adopted using mobile devices. However, little is known about how psychiatry residents use mobile technology, particularly mobile phones, in their training. The current data on how medical trainees use mobile devices as a part of their education are limited. A study from Canada has found that medical students receive instructions on searching and assessing primary medical literature, but few receive formal instruction on non-traditional tools such as general use search engines, Wikipedia, or social media [2]. The authors suggest that teaching about nontraditional sources may enhance the current curriculum. This was underscored by another survey of 62 psychiatry residents which showed that 68% primarily use online resources for education rather than printed materials [3]. Given that majority of the population use mobile apps to access digital media, it seems likely that medical students and residents also access clinical resources via their phones and apps [4]. However, it is unclear how residents and fellows in psychiatry use their mobile devices as a clinical resource. In 2014, Boruff et al [5] conducted a study to understand how Canadian medical students, residents, and faculty utilized their mobile phones to answer clinical questions and medical information. They found that mobile phones and tablets were broadly used in clinical settings and medical students were more likely to use these technologies. This is not a surprising finding due to the convenience and efficiency of mobile devices when retrieving information from the Internet. In a 2016 study, Gagnon et al [6] performed a systematic review of available literature to understand factors that influenced health care professionals in adapting mobile devices in their practice. They found that perceived usefulness and ease of use were some of the major factors in adapting mobile technologies among clinicians.

Although residents and fellows (trainees) are taught about protecting patient information in electronic medical records (EMRs), mobile technologies present new challenges that are distinct from those related to EMRs. Mobile devices like smartphones can be used interchangeably for professional and personal use including looking up patient information, sharing a photo on social media, and searching clinical decision support information. The use of these devices presents a new challenge to clinicians in integrating mobile phones in clinical use. These challenges include boundary issues as well as maintaining patient data safety. Although younger generations of psychiatry residents and fellows may often be considered tech savvy, their mobile phone use in clinical settings remains unclear.

To date, the most comprehensive data on mobile phone use in clinical practice are from a broad survey of physicians and nurses in the United Kingdom and psychiatry-specific data have not been reported [7]. Thus, in this study, we aim to provide an understanding on how psychiatric residents and fellows are using mobile phones in their clinical education and what barriers they perceive to this use.

## Methods

To understand how psychiatry residents and fellows use mobile technology in clinical practice, we performed a multisite survey in 2 psychiatry residency programs in different regions of the United States, which included Louisiana State University Health Sciences Center New Orleans, Louisiana, and Longwood psychiatry residency in Boston, Massachusetts. These surveys were approved by the institutional review board of each site, and the surveys were conducted anonymously and voluntarily. No compensation was provided to participate in the survey.

Paper surveys were provided to psychiatry trainees during grand rounds for a 4-week period. Surveys in New Orleans, Louisiana, were collected in May-June 2015, and then collected in Boston, Massachusetts, in November 2015. An electronic version of the survey on RedCap was available in New Orleans, Louisiana. There were a total of 26 questions on this survey, developed by the authors, which inquired about their mobile technology use in administrative tasks, communication, and treatment planning. Participants were also provided with text boxes to elaborate on apps they used in administrative tasks, communication, and treatment planning. We also surveyed residents on the possible barriers in implementing mobile technology in clinical practice through multiple choice as well as an option to write in barriers that were not included in the available choices. Barriers provided were based on a previous study that identified barriers in using electronic resources among medical students on their psychiatry clerkship [3]. The survey questions have been listed in Table 1. The completed surveys were subsequently entered and analyzed using Microsoft Excel on an encrypted and password-protected computer.

## Results

In New Orleans, 25 out of 41 trainees participated in this survey, and in Boston, 43 out of 52 trainees participated, which gave a total of 68 participants.

All the 68 participants owned a mobile phone or tablet. Moreover, 22 (32%) trainees practiced mostly in an outpatient setting and 41 (60%) practiced mostly in an inpatient setting. In addition, 5 (7%) trainees reported that they practice in a setting other than inpatient and outpatient psychiatry. A total of 54 (79%) of our participants reported that they use a mobile device to access protected patient information. Furthermore, 13 (19%) participants reported that they did not use a mobile device to access protected patient information, and 1 (1%) trainee did not respond to this question. Of the participants, 29 (43%) trainees reported using mental health-related apps to access protected patient information on their mobile device. Moreover, 40 (59%) trainees reported using an Internet site to access protected patient information.

A total of 16 (24%) trainees reported using a mobile device to communicate with patients, and 38 (56%) trainees reported that they did not use a mobile device to communicate with patients. Moreover, 14 (21%) participants did not respond when asked whether they used a mobile device to communicate with patients, and 38 (56%) trainees reported using a desktop computer to communicate with patients. Furthermore, 28 (41%) residents and fellows reported that they did not use a desktop computer to communicate with patients, and2 (3%) participants did not respond to this question. In addition, 26 (38%) trainees used a mobile device when implementing medication regimens, and 42 (62%) trainees did not use a mobile device when implementing medication regimens. A total of 16 (24%) participants reported using apps to implement medication regimens, and 15 (22%) trainees reported using an Internet site to implement medication regimens. Only 6 (9%) trainees reported using a mobile device to implement psychotherapy plans, and 61 (90%) trainees did not use a mobile device when implementing psychotherapy plans. Moreover, 1 (1%) trainee did not respond to this question. A total of 3 (4%) trainees used apps, and 2 (3%) trainees used Internet sites to implement psychotherapy plans.

Furthermore, 30 (44.1%) trainees used a mobile device to manage their clinic schedule; 38 (56%) trainees did not use a mobile device to manage their clinic schedule; 30 (44%) trainees intended to use a mobile device to manage their clinic schedule; and 38 (56%) trainees did not intend to use a mobile device to manage their clinic schedule.

**Table 1:** Survey questions.

Survey questions	Answer choices
How many years old are you?	_______ years old
Which one of the following genders do you most closely identify?	Male
	Female
	Transgender
	Prefer not to specify
Which one label do you most closely identify?	Attending physician
	Resident physician
	Medical student
	Other (please specify): _____________________
In which one practice setting do you spend most of your clinical hours?	Outpatient
	Inpatient
	Other
Do you own a smartphone or tablet?	Yes
	No
Do you intend to purchase a smartphone or tablet in the next 6 months?	Yes
	No
Do you use a mobile device (such as smartphone or tablet) to access protected patient information, such as their chart or their e-mail messages to you?	Yes
	No
Which methods do you use to access protected patient information on your mobile device? (Check all that apply)	Apps
	Internet Sites
	Other
Do you intend to use a mobile device to access protected patient information, such as their chart or their e-mail messages to you?	Yes
	No
Do you use a mobile device to communicate with patients?	Yes
	No
Do you use a desktop computer to communicate with patients?	Yes
	No
Do you use a mobile device when implementing medication regimens?	Yes
	No
Which methods do you use to implement medication regimens for your patient? (Check all that apply)	Apps
	Internet Sites
	Other
Do you use a mobile device when implementing psychotherapy plans?	Yes
	No
Which methods do you use to implement psychotherapy regimens for your patient? (Check all that apply)	Apps
	Internet Sites
	Other
Do you use a mobile device to manage your clinic schedule?	Yes
	No
Do you intend to use a mobile device to manage your clinic schedule?	Yes
	No
Do you use a mobile device to communicate with other staff?	Yes
	No
Which modality do you communicate with your mobile device? (Check all that apply)	Email
	Instant Messaging
	Text Messaging
	Call
	Other
In the last 3 months, have you recommended patients to use supplemental apps to their current medication management?	Yes
	No
Why or why not?	Text box
In the last 3 months, have you recommended patients to use supplemental apps to their current psychotherapy plan?	Yes
	No
Why or why not?	Text box
In the last 3 months, have you recommended online resources to patients?	Yes
	No
Why or why not?	Text box
What do you feel are the greatest barriers for using mobile devices in the care of patients? (Select up to three)	Privacy
	Safety
	Liability
	Cost
	Too much data
	Lack of evidence
	Lack of reimbursement
	Lack of clinical guidance

A total of 61 (90%) trainees used a mobile device to communicate with clinic staff and only 6 (9%) trainees did not. Moreover, 1 (1%) trainee did not answer this question. When asked about the modality used to communicate with clinic staff, 56 (84%) trainees reported using email, 10 (15%) trainees reported using an instant messaging app, 44 (66%) trainees reported using text, and 43 (64%) trainees reported calling clinic staff with their mobile devices.

In the last 3 months of completing the survey, 18 (27%) trainees reported recommending supplemental apps to patients in their current medication management, 48 (71%) trainees reported they do not, and 2 (3%) trainees did not respond to this question. Moreover, 19 (28%) trainees recommended supplemental apps to patients in their current psychotherapy plan, 48 (71%) trainees did not recommend any supplemental apps, and 1 (1%) trainee did not respond to this question. In the last 3 months of completing the survey, 38 (56%) trainees recommended online resources to patients, 27 (40%) trainees did not recommend online resources, and 3 (4%) trainees did not respond to this question.

When surveyed on perceived greatest barriers for using mobile devices in the care of patients, 38 (56%) trainees selected privacy, 27 (40%) trainees selected lack of clinical guidance, 20 (29%) trainees selected lack of evidence, 15 (22%) trainees selected liability, 12 (18%) trainees selected too much data, 11 (16%) trainees selected lack of reimbursement, 5 (7%) trainees selected cost, and 5 (7%) trainees selected safety.

Although the authors included questions on gender and age, there were a limited number of responses between both programs, and these questions were omitted in this study. Participants were provided with textboxes for various questions as listed in Table 1, but there were limited number of responses and they were omitted in this study. The above-described data are also provided in Tables 2 and 3.

**Table 2:** Survey responses.

Combined survey questions		n (%)
**In which** *one* **practice setting do you spend most of your clinical hours?**		
	Outpatient	22 (32.35)
	Inpatient	41 (60.3)
	Other	5 (7.4)
**Do you own a smartphone or tablet?**		
	Yes	68 (100)
	No	0 (0)
**Do you use a mobile device (such as a smartphone or tablet) to access protected patient information, such as their chart or their email messages to you?**		
	Yes	54 (79.41)
	No	13 (19.1)
	Did not answer	1 (1.5)
**Which modality do you communicate with your mobile device?**		
	Email	56 (83.5)
	Instant messaging app	10 (14.9)
	Text	44 (65.7)
	Call	43 (64.2)
**In the last 3 months, have you recommended patients to use supplemental apps to their current medication management?**		
	Yes	18 (26.5)
	No	48 (70.6)
	Did not answer	2 (2.9)
**In the last 3 months, have you recommended patients to use supplemental apps to their current psychotherapy plan?**		
	Yes	19 (27.9)
	No	48 (70.6)
	Did not answer	1 (1.5)
**In the last 3 months, have you recommended online resources to patients?**		
	Yes	38 (55.9)
	No	27 (39.7)
	Did not answer	3 (4.4)

**Table 3:** Perceived barriers by trainees.

Question	Privacy	Safety	Liability	Cost	Lack of evidence	Too much data	Lack of reimbursement	Lack of clinical guidance
What do you feel are the greatest barriers for using mobile devices in the care of patients? (Select up to three), n (%)	38 (55.9)	5 (7.4)	15 (22)	5 (7.4)	20 (29.4)	12 (17.6)	11 (16.2)	27 (39.7)

## Discussion

This multisite study provides the first results on both psychiatry trainee ownership and their use of mobile phones for clinical education and patient care. We found that all psychiatry residents who participated in this survey owned mobile phones, which exceeds the mobile phone usage in the general population. Further, the selected programs cover multiple hospitals within their city and are exposed to various types of EMRs. Although psychiatry residents own mobile phones, their reported use in our survey suggests that it is limited. The most common reported clinical use is communicating with clinical staff, especially for scheduling, and the second most common use is to access patient information. Although these uses may seem simple in that mobile phones are being used for communication, each also raises educational opportunities for educators to consider. There have been recent efforts to ensure residents are educated about best practices with social media and websites such as Facebook, and in 2014, Dejong and Gorrindo [8] have discussed about texting and the professionalism principles when communicating with patients using this method. Among internal medicine residents, the use of short message service (SMS) texting as a means of communication has also raised the ethical question of whether the ease of use is a potential breach of patient privacy [9]. Overall, there remains a limited amount of literature on how we teach psychiatry residents best practices for using mobile devices in clinical care roles. If residents are using them primarily for scheduling purposes, it is not difficult to imagine that soon they will also be using mobile phones for direct clinical care roles as well – and indeed our survey suggests that some already are.

Several limitations of this study should be noted. All data were self-reported, and we did not verify through direct observation how residents are using mobile phones and apps in clinical care. Although our study is the first to examine this topic, our questions were not exhaustive and did not include details on app use when using with patients and communicating with other staff members. We attempted to collect demographic data including gender and age, but due to low compliance, the limited data gathered could not be analyzed. We also did not obtain details on how residents evaluate and consider whether to use or not use an app. We designed a quantitative design with yes/no questions to understand the basics of mobile device usage among trainees. Future iterations of our study with Likert scales and more qualitative metrics would be important to complete the understanding of mobile device use among trainees. Due to concerns of lack of participation, we also designed our survey with yes/no questions to help increase compliance. It is important to note that our study was limited to 2 sites, and it is difficult to assess how generalizable our results are outside of these 2 study sites.

Given this was a convenience sample and we have no information from the residents who did not choose to participate, it is, however, possible that the residents more interested in technology were more likely to participate. On the basis of our survey, future studies can consider larger trainee population of various specialties, while providing more flexibility in the survey including clarifying practice setting and providing more write in options. This can help provide further data and understanding on which areas of practice should be targeted through education as well as enhancing work flow using technology.

Despite a robust app development industry, clinical evidence for the effectiveness of apps in psychiatry remains nascent. Further, evaluating the safety and quality of apps is challenging. Thus, the fact that some residents are venturing into this largely unknown space of apps is notable. Generally, our survey suggests that trainees are at times hesitant in using mobile devices. However, a partial integration in their clinical practice is evident in that over 89% of trainees use their mobile devices to communicate with clinical staff. In addition, over 55% report using desktop computers to communicate with their patients, and the authors expect trainees to transition to using mobile devices in their patient communication due to increased availability and convenience of mobile devices. This reflects the need for educators and supervisors to consider at least inquiring if residents are using these tools and to prepare our trainees to potentially utilize these tools in practice.

**Table 4:** Suggested curriculum – Technology in Psychiatry Seminar (TIPS).

Topic	Description
“Anatomy” and “Physiology”	To understand basic inner works of technologies available to medical professionals and patients – includes understanding of basic technology terminology, how technologies are developed, and types of technology that are currently used in psychiatry
Telepsychiatry 1	Review literature in telepsychiatry Understand pros and cons of telepsychiatry
Telepsychiatry 2	Review of cases in telepsychiatry including tips to be used in practice Review basic video conferencing etiquette
Mobile technology	Review of current literature on mobile technology use in various psychopathology – mood disorder, anxiety, sleep, etc Discuss current applications of mobile technology
Professionalism, ethics, and privacy	To discuss important boundaries between the digital doctor–patient relationship, including texting/emailing etiquette, professional social media use, and maintaining a professional presence online Discuss maintenance of patient privacy when using technology
Research	Understand basic research study design in technology and review of current literature
Technology and psychopathology	Psychopathology relating to technology: Internet gaming disorder, Internet addiction disorder, Online gaming industry etc Using technology to treat psychopathology – mindfulness, CBT, and CBTi
Counseling patients on safe technology use	How to counsel patients on safe technology use – sleep hygiene, self-evaluation of apps, making sure it is a licensed clinician on telepsychiatry platform, etc

Along these lines, our survey results suggest several opportunities for psychiatric educators to offer guidance and support for trainees regarding the use of mobile technology. Residents raised several important concerns about apps including privacy, lack of clinical guidance, lack of evidence, and liability, among others. However, each of these topics represents a complex area as mobile technology for health care is advancing more rapidly than legislation, digital security, clinical trials, and clinical teaching can equal. Another challenge is that many educators may be less familiar or excited by mobile phone apps and other new technologies and thus lack the necessary experiences to educate residents. Given that “lack of clinical guidance” was selected as the number 2 barrier to using mobile technology in patient care, this suggests there is a need for a curriculum focused on digital and mobile technologies. Creating a curriculum in technology during graduate medical training could provide a platform for introducing trainees to basic concepts about current technologies including mobile technology and technology use in patient care [10]. In a technology curriculum, the core concept is not only to include basic concepts but also to provide principles for evaluating technology from a clinical perspective. Therefore, the authors propose the development of a technology curriculum that educates trainees in various digital psychiatry tools including EMR, telepsychiatry, mobile device use, and wearables. This potential technology curriculum is listed in Table 4. Further research and experience would be needed to find the most effective curriculum. Fundamentally, having a technology curriculum can allow trainees to better understand technologies relating to patient care without over incorporation in daily practice.

From this study, trainees are interested in using and have used mobile technology in practice. However, their education on how to use it remains lacking, and educators should consider including mobile technology education as a part of the residents’ curriculum. Encouraging mobile technology research is also important. Until further research is conducted and empirical data are collected, basic clinical pearls and core concepts for mobile technology such as understanding the clinical evidence and privacy regulations could be the foundation of a technology curriculum. Although research- and evidence-based use of mobile technology is still in development, educators can teach residents how to dissect an app beyond its appearance and usability. Currently, The American Psychiatric Association’s App Evaluation Task Force Committee is trying to understand mobile technology and create guidelines for clinicians to better evaluate apps for themselves and for patients [11].

Psychiatry trainees use mobile phones for their work, mainly for scheduling and administrative tasks. Though they remain hesitant to incorporate mobile health into their direct clinical care, partial integration of a mobile device is evident. Part of the hesitance among trainees appears to be due to the lack of education and guidance during their current training. It is imperative to prepare trainees to practice in the 21st century where mobile phone use is part of daily life so that they will be knowledgeable in helping patients understand potential impacts of mobile technology. Such preparation and training may require the development of a new curriculum and educational efforts.

## Abbreviations

CBT: Cognitive Behavioral Therapy
CBTi: Cognitive Behavioral Therapy for insomnia
EMRs: electronic medical records
SMS: electronic medical records
TIPS: Technology in Psychiatry Seminar
